# Mass Spectrometry Evaluation of Biomarkers in the Vitreous Fluid in Gaucher Disease Type 3 with Disease Progression Despite Long-Term Treatment

**DOI:** 10.3390/diagnostics10020069

**Published:** 2020-01-26

**Authors:** Aizeddin Mhanni, Michel Boutin, Frank Stockl, Janine Johnston, Jeff Barnes, Donald Duerksen, Leanne Zimmer, Christiane Auray-Blais, Cheryl Rockman-Greenberg

**Affiliations:** 1 Department of Pediatrics and Child Health, University of Manitoba, Winnipeg, MB R3T2N2, Canada; amhanni@hsc.mb.ca; 2 Department of Biochemistry and Medical Genetics, University of Manitoba, Winnipeg, MB R3T2N2, Canada; 3 Department of Pediatrics, Université de Sherbrooke, Sherbrooke, QC J1H 5N4, Canada; Michel.Boutin2@USherbrooke.ca (M.B.); Christiane.Auray@USherbrooke.ca (C.A.-B.); 4 Department of Ophthalmology, University of Manitoba, Winnipeg, MB R3T2N2, Canada; fstockl@icloud.com (F.S.); jjohnston@ciads.ca (J.J.); 5 Department of Medicine, University of Manitoba, Winnipeg, MB R3T2N2, Canada; jbarnes1@hsc.mb.ca (J.B.); dduerkse@sbgh.mb.ca (D.D.); 6 Manitoba Association of Optometrists, Winnipeg, MB R3H0Y4, Canada

**Keywords:** Gaucher, LSD, glucosylceramide, UPLC-MS/MS, vitreous

## Abstract

Intraocular lesions have been infrequently reported in patients with Gaucher disease type 3 (GD3). We previously reported siblings with GD3 who responded well to the combination of enzyme replacement therapy (ERT) and substrate reduction therapy (SRT). Here we report progressive bilateral vitreous and preretinal deposits with declining visual acuity requiring bilateral vitrectomies in one of these siblings. These ocular manifestations had progressed despite combined ERT and SRT with improvement in visual acuity after vitrectomies. Vitrectomy fluid analysis performed for the first time by ultra-performance liquid chromatography–tandem mass spectrometry (UPLC-MS/MS) identified a high concentration of glucosylceramide (GluCer) in the patient (262.842 nM) compared to a sample (0.428 nM from a patient without a lysosomal storage or known hereditary metabolic disorder). The GluCer detected in our patient was resolved into 12 different isoforms including two methylated ones. No evidence of galactosylceramide (GalCer) was detected. The development of these intraocular manifestations and their characterization by UPLC-MS/MS indicate a need for ongoing ophthalmologic evaluation of all GD patients and for new therapies that can cross the blood–retinal and blood–brain barriers for patients with GD and other neuropathic lysosomal storage disorders.

## 1. Introduction

Gaucher disease (GD) (OMIM #230800) is an autosomal recessive lysosomal storage disorder (LSD) caused by *GBA1* gene mutations and resultant decreased activity of β-glucosidase (glucocerebrosidase) (GCase) [1]. This latter enzyme cleaves the glucose moiety of glucosylceramide (GluCer) and glucosylsphingosine (GluSph) involved in sphingolipid catabolism. The GluCer and GluSph accumulations in biological fluids and reticuloendothelial cells lead to varying multisystem and visceral involvement now considered to be on a continuum of disease historically classified as GD types 1, 2 and 3 [1,2]. The incidence of GD ranges between 1/40,000 and 1/60,000 [2]. Gaucher disease type 3 (GD3) is overrepresented in patients from northern Sweden with a prevalence of 1/17,500, in large part due to the *GBA1* L444P (c.1448 T>C) mutation [3]. Approximately 5% of Gaucher patients in the West belong to the type 3 subset, also called juvenile or subacute neurological GD (GD3), which is associated with variable neurological involvement including horizontal supranuclear gaze palsy, strabismus, epilepsy, ataxia, polyneuropathy, Parkinsonism, and cognitive impairment [4]. El-Beshlawy et al. (2017) summarized the phenotypic, demographic, and genotypic characteristics of 253 patients with GD3 enrolled in the global ICGG Gaucher Registry [5]. GD progression has been documented by ourselves and others despite treatment with enzyme replacement therapy (ERT) or substrate reduction therapy (SRT) [6,7,8]. 

Winter et al. (2019) have recently published an extensive review of the ocular findings in patients with all types of GD [9]. Ocular preretinal deposits have previously been reported in only ~3% of 80 patients with GD1 [2]. In GD3 patients, reports of ocular manifestations are also infrequent and include corneal opacification and pinguecula, cherry red maculae, and retinal lesions typical of the preretinal accumulation of glycolipids [10,11,12]. Some reports document progression of vitreous opacities despite ERT [8,13]. Such GD progression despite treatment with ERT or SRT has been attributed to antibody formation, lack of penetration of enzyme, or progressive natural history of disease. We now report severe intraocular involvement in a 20-year-old patient with GD3 despite long-term treatment with ERT and SRT and, for the first time, the vitreous fluid of such a GD3 patient was analyzed by UPLC-MS/MS. These deposits were confirmed to be exclusively different isoforms of GluCer, a substrate for β-glucosidase. 

## 2. Materials and Methods

### 2.1. Clinical Case Report

We present a 20-year-old male with GD3 due to homozygous *GBA1* L444P mutations. He has mild stable neurologic impairment with minimal tremor, is of normal intelligence, and has a normal brain MRI. The patient has been treated with imiglucerase (Sanofi-Genzyme Corporation, Cambridge, MA, USA) since the age of 18 months. At age 10 years, miglustat (Actelion Pharmaceuticals LT, Allschwil, Switzerland) was added to his treatment regime due to pulmonary and bony progression, reported in Mhanni et al. (2016) [7]. Ophthalmologic examination at age 14 years showed blink-saccade synkinesis and normal fundi. At age 16 years, the patient developed severe protein-losing enteropathy (PLE), malnutrition, and malabsorption with calcified peritoneal lymph nodes [7]. PLE stabilized with total parenteral nutrition including amino acids, glucose, and lipid (~1 g/kg/day), as well as oral medium-chain triglyceride oil supplements, oral low-dose budesonide, and a low-fat and disaccharide-free diet. ERT and SRT at recommended therapeutic dosages were continued [7]. At this time, he was also noted to have scattered vitreous and preretinal white deposits which were asymptomatic. 

**Figure 1:**
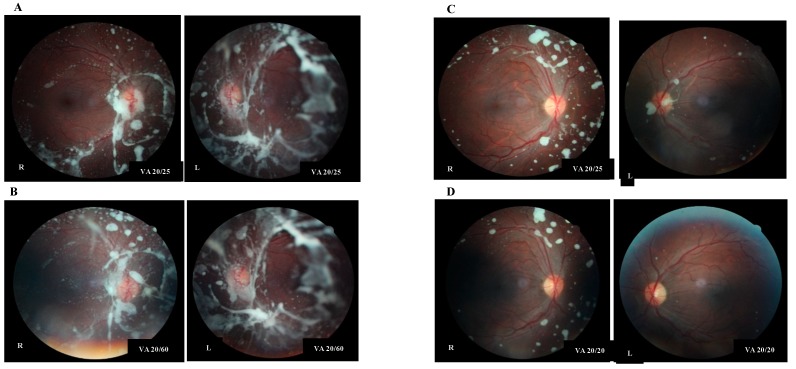
Preoperative fundus photographs showing progression of the deposits and decreasing visual acuity in the right (R) and left (L) eyes (**A** and **B**). Postoperative fundus photographs showing reduction in deposits in both eyes (**C**) and improvement in visual acuity (**D**).

At age 18 years, he complained of floaters which he described as seeing “orbs” throughout his visual field. His visual acuity was measured at 20/25 in the right eye and 20/20 in the left eye. Eight months later he complained of a gradual decline in vision in his left eye. Examination revealed visual acuity of 20/25 in the right eye and 20/60 in the left eye. Fundus examination revealed increased lipid deposits, peripapillary vitreomacular traction with associated epiretinal membranes in both eyes (Figure 1A,B). Pars plana vitrectomy was performed on his left eye four months after the decline in his vision in the left eye. Vision declined to 20/60 in the right eye three months later and pars plana vitrectomy was performed on the right eye. 

A standard 25G, three-port pars plana vitrectomy was performed on both eyes. Surgical intervention consisted of detachment of the posterior hyloid, removal of lipid deposits and attempted aspiration of lipid deposits on the retinal surface, and epiretinal and internal limiting membrane peeling. Some preretinal deposits were extremely adherent to the retina and, as a result, some residual deposits remained at the conclusion of surgery. There was significant improvement in the lipid deposits and significant improvement of structural anatomy of the macular anatomy post operatively (Figure 1C,D). Visual acuity improved to 20/20 in both eyes.

A vitreous fluid sample from a control patient not affected with an LSD was obtained. The patient was undergoing a vitrectomy for epiretinal membranes post trauma. 

All procedures followed were in accordance with the ethical standards of the responsible committee on human experimentation (institutional and national) and with the Helsinki Declaration of 1975, as revised in 2000. Informed consent was obtained from the patient (and his family) reported in this manuscript and the patient agrees with the content and with submitting this paper for consideration for publication.

### 2.2. Experimental Section

#### 2.2.1. Vitrectomy Fluid Samples

The volume of irrigated and aspirated fluid contents from the vitrectomy of the GD3 patient and control totaled 100 mL and 50 mL, respectively. Both specimens were kept frozen at −20 °C until analysis. 

#### 2.2.2. Reagents

The GluCer isoform mixture from the Gaucher spleen (˃98%), galactosylceramide (GalCer) isoform mixture (˃98%), and GluCer(C16:0)D_3_ were purchased from Matreya (Pleasant Gap, PA, USA). Optima LC/MS grade water and A.C.S. reagent grade ammonium formate (amm. form.) were from Fisher Scientific (Fair Lawn, NJ, USA). HPLC grade methanol (MeOH) and acetonitrile (ACN), and A.C.S. grade acetone were supplied by EMD Chemicals Inc. (Darmstadt, Germany). ReagentPlus grade dimethyl sulfoxide (DMSO) (≥99.5%) and terfenadine were from Sigma-Aldrich (St. Louis, MO, USA) and formic acid (FA) (˃99%) from Acros Organics (Morris Plain, NJ, USA).

#### 2.2.3. Sample Preparation

The vitrectomy fluid specimens were purified using hydrophilic-lipophylic balance (HLB) solid-phase extraction cartridges (30 mg, Oasis, Waters Corp., Milford, MA, USA) using a method adapted from Hamler et al. [14]. Briefly, 1 mL of vitrectomy fluid was diluted with 1830 µL of MeOH, 1042 µL of acetone, 105 µL of DMSO, and 20 µL of GluCer(C16:0)D_3_ (10 µmol/L in DMSO) and loaded on the solid-phase extraction cartridge preconditioned with 1 mL of MeOH and 1 mL of water, respectively. The cartridge was then washed with 2 mL of (60% MeOH/30% H_2_O/10% acetone) and the sample was eluted with 2 mL of (90% acetone/10% MeOH). The specimen was evaporated to dryness using a nitrogen stream and resuspended in 100 µL of (20% DMSO/80% (94.5% ACN/2.5% MeOH/2.5% H_2_O/0.5% FA/ 5 mM (amm. form.)).

#### 2.2.4. UPLC-MS/MS Analysis of GluCer Isoforms in the Vitrectomy Fluid

The ultra-high-performance liquid chromatography (UPLC) coupled to tandem mass spectrometry (MS/MS) method for the analysis of GluCer isoforms in the vitrectomy fluid was adapted from Boutin et al. [15]. The sample purification was performed on an Acquity H-Class UPLC system using a Halo Hilic 2.7 column (4.6 × 150 mm, particles 2.7 µm, Advanced Materials Technology, Wilmington, DE, USA). The mobile phase was (94.5% ACN/2.5% MeOH/2.5% H_2_O/0.5% FA/5 mM amm. form.) and the strong and weak wash solutions were 100% ACN. The elution was isocratic at a flow rate of 0.5 mL/min and the injection volume was 5 µL. The mass spectrometry analyses were performed on a Xevo TQ-S Micro from Waters Corp. using the multiple reaction monitoring mode. For the positive electrospray ionization (ESI+), the capillary, sampling cone, and source offset voltages were 3.0 kV, 10 V, and 50 V, respectively; the source and desolvation temperatures were 120 °C and 350 °C, respectively. The desolvation and cone gas flows were 1000 L/h and 150 L/h, respectively. For all 30 GluCer isoforms analyzed, the fragment corresponding to the di-dehydrated sphingosine moiety (*m/z* 264.3) was analyzed. The dwell time was 0.1 s and the collision energy was 32 V. The GluCer isoform abundances were determined by comparison with the signal of the GluCer(C16:0)D3 (0.2 µM) internal standard contained in the injected samples. 

#### 2.2.5. UPLC-Q-Tof-MS Analysis of GluCer(C16:0)

A fragmentation study of the C16:0 isoform of GluCer contained in the GD3 vitreous fluid was performed on a quadrupole time-of-flight (Q-tof, Synapt G1, Waters Corp.) mass spectrometer. For the positive electrospray ionization (ESI+), the capillary, sampling cone, and the extraction cone voltages were 3.2 kV, 10 V, and 4 V, respectively; the source and desolvation temperatures were 120 °C and 450 °C, respectively; the desolvation, cone gas, and trap gas flows were 700 L/h, 30 L/h, and 1.5 mL/min, respectively. A collision energy ramp of 5–35 V was used. The real time recalibration (lock-mass) was performed by infusing a terfenadine solution of 31.25 nM (5% ACN, 0.2% FA). The following parameters for the lock-mass probe were used: scan time: 0.5 s, intervals 5 s, sampling cone voltage: 6 V, analyzed mass: 472.3215 Da, mass window: ± 0.2 Da, and scan average: 3. 

### 2.3. Patient Consent Statements

Patients provided full consent for publication including images.

## 3. Results 

The presented UPLC-MS/MS method allowed the baseline separation between GluCer isoforms and their GalCer structural isomers (Supplemental Figure S1). Care was taken to separate the GluCer isoforms from their GalCer isobaric interferences. The two structural isomers are differentiated only by the axial or equatorial conformation of one hydroxyl group. GalCer is not a substrate of β-glucosidase and is known to be more abundant than GluCer in brain tissues. A total of 12 GluCer isoforms, including two methylated ones, were detected in the vitrectomy fluid of the GD3 patient among the 30 GluCer isoforms analyzed. The high number of isoforms detected is due to the presence of diverse fatty acid chains in the ceramide moiety [15]. The total amount of GluCer in the vitrectomy fluid corresponds to the measured concentration of GluCer multiplied by the total volume of vitrectomy fluid (Table 1). 

**Figure 2:**
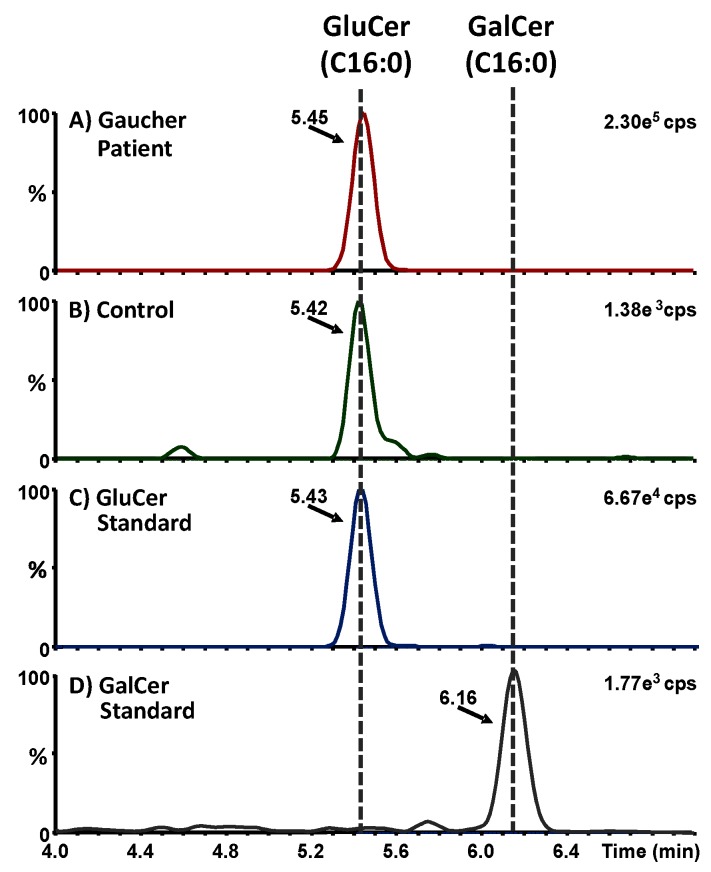
Multiple reaction monitoring (MRM) analysis of GluCer(C16:0) and GalCer(C16:0) (*m/z* 700.57 > 264.27) in (**A**) vitrectomy fluid from the Gaucher type 3 patient; (**B**) vitrectomy fluid from a control; (**C**) commercial standard mixture of GluCer isoforms; and (**D**) commercial standard mixture of GalCer isoforms. Dotted lines correspond to mean retention times for GluCer(C16:0) and GalCer(16:0). Cps, counts per second.

**Figure 3:**
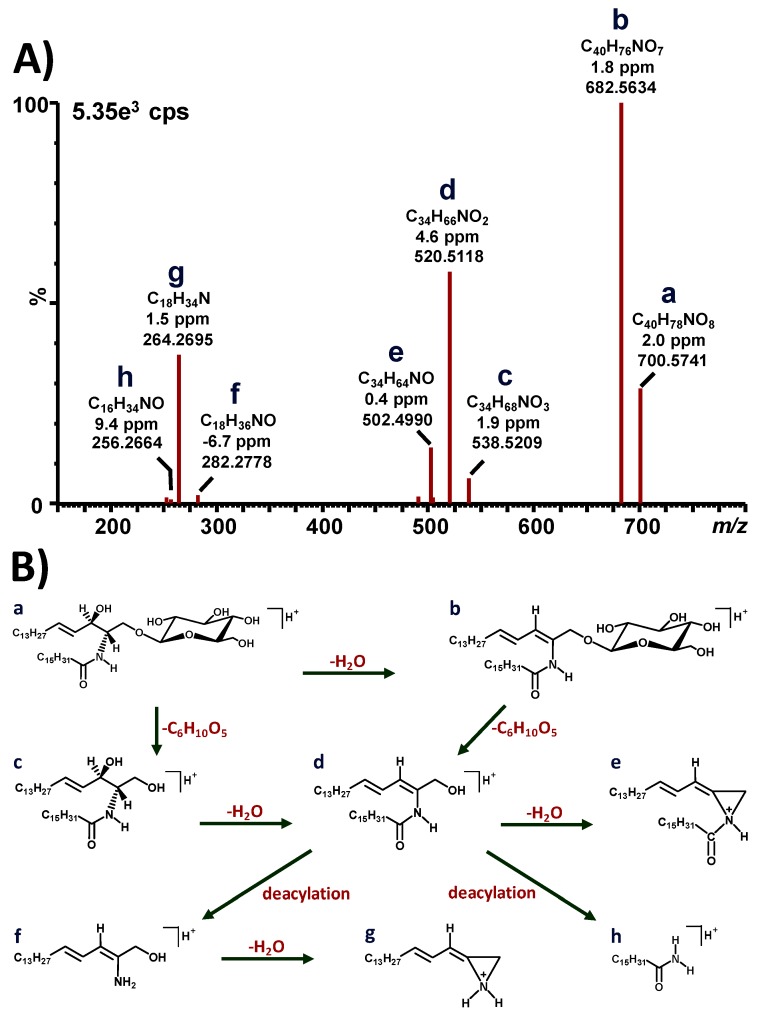
(**A**) Quadrupole time-of-flight (Q-tof) fragmentation spectrum of GluCer(C16:0) (*m/z* 700.57) in vitreous humor of the Gaucher type 3 patient. Deviations from the theoretical masses written in parts per million (ppm). Collision energy ramp: 5–35 V. Cps, counts per second. (**B**) Fragmentation mechanism of GluCer(C16:0) corresponding to the spectrum in (**A**).

Similar methylation of the amide linkage was previously observed on globotriaosylceramide (Gb_3_) [16,17] and galabiosylceramide (Ga_2_) [18], which are other glycosphinglipids structurally related to GluCer and which accumulate in Fabry disease. The total concentration of GluCer isoforms in the vitreous fluid of our GD3 was more than 1250 times the levels of the control. Only the C16:0 GluCer isoform was detectable in the vitrectomy fluid of the control. Among the 12 GluCer isoforms detected in our GD patient, 10 of these isoforms were confirmed by retention time comparison with a commercial standard. Figure 2A,B shows the ion chromatograms of GluCer(C16:0) in the vitrectomy fluid of the GD3 patient and the control, respectively. Figure 2C,D shows the ion chromatograms of GluCer(C16:0) and GalCer(C16:0) with a clear separation between the two structural isomers. Moreover, the concentration of the GluCer(C16:0) isoform was sufficiently high in the vitrectomy fluid of the GD3 patient to validate its structure by tandem mass spectrometry using a quadrupole/time-of-flight instrument. Figure 3A shows the fragmentation spectrum obtained for the GluCer(C16:0) isoform detected in the vitrectomy fluid of the Gaucher patient, while Figure 3B shows its fragmentation mechanism. Mass errors ≤9.4 ppm were measured for the different GluCer(C16:0) fragments. Cholesterol and triglycerides were not detected. Histologic analysis of the fluid was not performed. Glucosylsphingosine (lyso-Gb_1_) concentrations in body fluids, including the vitreous fluid, were not monitored in this patient since it was not part of routine clinical care at the time.

## 4. Discussion

We report bilateral, diffusely distributed, and progressive deposits interfering with vision in a patient with GD3 despite 18 years of ERT and 10 years of SRT. We characterized the biomarkers in the vitrectomy fluid of the patient for the first time by UPLC-MS/MS and found an excess of GluCer isoforms. We identified six previous reports of vitreous and preretinal deposits in patients with GD1 and GD3 [8,11,13,19,20,21]. To our knowledge, four of these patients received ERT or SRT [13,18,19,21]. Shrier et al. (2004) analyzed the vitreous fluid from a GD patient by thin layer chromatography (TLC) [19]. Their results show large amounts of GluCer without identifying the various isoforms. Our mass spectrometry methodology allowed a more selective and quantitative UPLC-MS/MS analysis of several isoforms of GluCer in the vitreous fluid of our GD3 patient. One 20-year-old female with GD3 and vitreous opacities similar to our patient was previously reported [8]. She had developed opacities despite receiving ERT for over 10 years. The vitreous opacities were most prominent in the periphery and there were bilateral epiretinal membranes (ERMs) [8]. In this case, optical coherence tomography was performed pre-, post-, and post-second vitrectomy. The pre-image illustrated retinal contour distortion due to the ERMs. The post-vitrectomy image demonstrated that the ERMs will rapidly return if the internal limiting membrane is not removed during the vitrectomy. Interestingly, in response to vitrectomy, our patient’s vision improved to 20/20 with further clearing of the retinal opacities post-vitrectomies. 

Our patient’s affected older sister, also on long-term ERT and SRT, exhibits scattered white dots preretinally only adjacent to retinal vessels. These have not progressed in over five years and have not interfered with her vision. Of note, neither of the siblings had a splenectomy. The origin of the material, the progressive improvement in appearance of the deposits and in visual acuity postoperatively in our patient, and the reason for the discordance between affected siblings are uncertain. Since the deposits appear to be perivascular in the sister, this suggests that the deposits may be vascular in origin. Seidova et al. (2009) noted the deposits seen in their patient’s eyes were positioned on a blood vessel similar to our patient’s sister [21]. One explanation that was proposed as to why these deposits appear despite the use of ERT is that ERT is unable to cross the blood–retinal barrier and blood–brain barrier (BBB) [21]. Miglustat, which crosses the BBB, is indicated for the treatment of GD1. However, effectiveness is limited; its use is therefore restricted to adults with mild to moderate GD1 and those with GD1 who cannot tolerate ERT or show disease progression [22]. Our patient and his sibling were on SRT with miglustat until two years ago. This was stopped by the patients due to gastrointestinal side effects. We note that vitreous fluid contains cells called hyalocytes which are felt to be derived from monocyte/macrophage lineage but not from retinal cells such as glial cells or retinal pigment epithelium [23]. Thus, it is possible that these GD lipid deposits may also originate in these hyalocytes. Unfortunately histologic analysis of the vitreous aspirates was not performed in our patient. Future perspectives include the evaluation of various ceramides and glucosylsphingosine for both siblings in order to better understand this complex disease. 

## 5. Conclusions

We report a patient with GD3 who underwent bilateral vitrectomies to remove white vitreous and preretinal deposits of GluCer that developed despite long-term treatment with combined ERT and SRT. To our knowledge, it is the first time that GluCer isoforms were analyzed in the vitreous fluid from a GD3 patient and unaffected control using UPLC-MS/MS, and the relative quantitation of various isoforms is provided. After the vitrectomy, the visual acuity improved in both eyes of our patient and is now 20/20, stable 2.5 years postoperatively. The development of these intraocular manifestations indicates a need for ongoing ophthalmologic evaluation of all GD patients and the need for new therapies that can cross the blood–retinal and blood–brain barriers for patients with GD and neuropathic LSD.

## Figures and Tables

**Table 1:** Retention time and concentration of 30 glucosylceramide (GluCer) isoforms measured by ultra-high-performance liquid chromatography–tandem mass spectrometry (UPLC-MS/MS) in vitrectomy fluids from a pathological control and from a Gaucher type 3 patient.

		Concentration in Vitrectomy Fluid		Total Amount in Vitrectomy Fluid	
Biomarker	Retention Time	Control (50 mL)	Gaucher (100 mL)	Control	Gaucher
	Min	nM	nM	nmol	Nmol
GluCer(C16:0)*	5.45	0.428	100.489	0.021	10.049
GluCer(C18:0)*	5.24	nd	11.860	nd	1.186
GluCer(C20:0)*	5.14	nd	7.627	nd	0.763
GluCer(C22:0)*	5.01	nd	20.548	nd	2.055
GluCer(C24:0)*	4.86	nd	32.827	nd	3.283
**GluCer(C26:0)**	4.77	nd	0.749	nd	0.075
**GluCer(C16:1)**	Nd	nd	nd	nd	Nd
**GluCer(C18:1)**	Nd	nd	nd	nd	Nd
**GluCer(C20:1)**	Nd	nd	nd	nd	Nd
GluCer(C22:1)*	5.05	nd	5.238	nd	0.524
GluCer(C24:1)*	4.95	nd	75.266	nd	7.527
**GluCer(C26:1)**	4.85	nd	4.270	nd	0.427
**GluCer(C22:2)**	Nd	nd	nd	nd	Nd
GluCer(C24:2)*	4.99	nd	1.099	nd	0.110
GluCer(C16:0)Me*	5.32	nd	1.772	nd	0.177
**GluCer(C18:0)Me**	Nd	nd	nd	nd	Nd
**GluCer(C20:0)Me**	Nd	nd	nd	nd	Nd
GluCer(C22:0)Me*	4.92	nd	1.097	nd	0.110
**GluCer(C24:0)Me**	Nd	nd	nd	nd	Nd
**GluCer(C26:0)Me**	Nd	nd	nd	nd	Nd
**GluCer(C22:1)Me**	Nd	nd	nd	nd	Nd
**GluCer(C24:1)Me**	Nd	nd	nd	nd	Nd
**GluCer(C16:0)OH**	Nd	nd	nd	nd	Nd
**GluCer(C18:0)OH**	Nd	nd	nd	nd	Nd
**GluCer(C20:0)OH**	Nd	nd	nd	nd	Nd
**GluCer(C22:0)OH**	Nd	nd	nd	nd	Nd
**GluCer(C24:0)OH**	Nd	nd	nd	nd	Nd
**GluCer(C26:0)OH**	Nd	nd	nd	nd	Nd
**GluCer(C22:1)OH**	Nd	nd	nd	nd	Nd
**GluCer(C24:1)OH**	Nd	nd	nd	nd	Nd
**Total**	na	0.428	262.842	0.021	26.286

* Molecule identification confirmed by retention time comparison with a commercial GluCer standard mixture. Nd, not detected; na, not applicable.

## Supplementary Materials

The following are available online at https://www.mdpi.com/2075-4418/10/2/69/s1, Figure S1: Structures of (**A**) glucosylceramide (GluCer) and (**B**) galactosylceramide (GalCer) with a C22:0 fatty acid. The glucocerebrosidase (GCase) cleavage site is indicated with scissors on the GluCer molecule. Dotted circles indicate the hydroxyl groups differentiating GluCer and GalCer isomers by their axial or equatorial conformations.

## References

[1] Ferreira C.R., Gahl W.A. (2017). Lysosomal storage diseases. Transl. Sci. Rare Dis..

[2] Alaei M.R., Tabrizi A., Jafari N., Mozafari H. (2019). Gaucher Disease: New Expanded Classification Emphasizing Neurological Features. Iran. J. Child. Neurol..

[3] Machaczka M., Paucar M., Björkvall C.K., Smith N.J.C., Cox T.M., Forsgren L., Svenningsson P. (2018). Novel hyperkinetic dystonia-like manifestation and neurological disease course of Swedish Gaucher patients. Blood Cells Mol. Dis..

[4] Stirnemann J., Belmatoug N., Camou F., Serratrice C., Foissart R., Caillaud C., Levade T., Astudillo L., Serratrice J., Brassier A. (2017). A Review of Gaucher Disease Pathophysiology, Clinical Presentation and Treatments. Int. J. Mol. Sci..

[5] El-Beshlawy A., Tylki-Szymanska A., Vellodi A., Belmatoug N., Grabowski G., Kolodny E., Batista J., Cox G., Mistry P. (2017). Long-term hematological, visceral, and growth outcomes in children with Gaucher disease type 3 treated with imiglucerase in the International Collaborative Gaucher Group Gaucher Registry. Mol. Genet. Metab..

[6] Burrow T.A., Sun Y., Prada C.E., Bailey L., Zhang W., Brewer A., Wu S.W., Setchell K.D.R., Witte D., Cohen M.B. (2015). CNS, lung, and lymph node involvement in Gaucher disease type 3 after 11 years of therapy: Clinical, histopathologic, and biochemical findings. Mol. Genet. Metab..

[7] Mhanni A.A., Kozenko M., Hartley J.N., Deneau M., El-Matary W., Rockman-Greenberg C. (2016). Successful therapy for protein-losing enteropathy caused by chronic neuronopathic Gaucher disease. Mol. Genet. Metab. Rep..

[8] Storey P.P., Tan J.J., Rayess N., Rao N. (2018). Bilateral Pars Plana Vitrectomy for Vitreous Opacities and Epiretinal Membrane in Gaucher Disease. J. VitreoRetinal Dis..

[9] Winter A.W., Salimi A., Ospina L.H., Roos J.C.P. (2019). Ophthalmic manifestations of Gaucher disease: The most common lysosomal storage disorder. Br. J. Ophthalmol..

[10] Seehra G.K., Eghbali A., Sidransky E., FitzGibbon E. (2020). White vitreous opacities in five patients with Gaucher disease type 3. Am. J. Med. Genet. A.

[11] Cogan D.G., Chu F.C., Gittinger J., Tychsen L. (1980). Fundal abnormalities of Gaucher’s disease. Arch. Ophthalmol..

[12] Abrahamov A., Elstein D., Gross-Tsur V., Farber B., Glaser Y., Hadas-Halpern I., Ronen S., Tafakjdi M., Horowitz M., Zimran A. (1995). Gaucher’s disease variant characterised by progressive calcification of heart valves and unique genotype. Lancet.

[13] Watanabe A., Gekka T., Arai K., Tsuneoka H. (2017). A case of traction retinal detachment in a patient with Gaucher disease. Ophthalmic Genet..

[14] Hamler R., Brignol N., Clark S.W., Morrison S., Dungan L.B., Chang H.H., Khanna R., Frascella M., Valenzano K.J., Benjamin E.R. (2017). Glucosylceramide and Glucosylsphingosine Quantitation by Liquid Chromatography-Tandem Mass Spectrometry to Enable In Vivo Preclinical Studies of Neuropathic Gaucher Disease. Anal. Chem..

[15] Boutin M., Sun Y., Shacka J.J., Auray-Blais C. (2016). Tandem Mass Spectrometry Multiplex Analysis of Glucosylceramide and Galactosylceramide Isoforms in Brain Tissues at Different Stages of Parkinson Disease. Anal. Chem..

[16] Auray-Blais C., Boutin M. (2012). Novel Gb_3_ isoforms detected in urine of Fabry disease patients: A metabolomic study. Curr. Med. Chem..

[17] Manwaring V., Boutin M., Auray-Blais C. (2013). A metabolomic study to identify new globotriaosylceramide-related biomarkers in the plasma of Fabry disease patients. Anal. Chem..

[18] Boutin M., Auray-Blais C. (2015). Metabolomic discovery of novel urinary galabiosylceramide analogs as Fabry disease biomarkers. J. Am. Soc. Mass. Spectrom..

[19] Shrier E.M., Barr C.C., Grabowski G.A. (2004). Vitreous opacities and retinal vascular abnormalities in Gaucher disease. Arch. Ophthalmol..

[20] Hsing Y.E., Foster A. (2014). Preretinal and posterior vitreous deposits in Gaucher disease. JAMA Ophthalmol..

[21] Seidova S.F., Kotliar K., Foerger F., Klopfer M., Lanzl I. (2009). Functional retinal changes in Gaucher disease. Doc. Ophthalmol..

[22] Martín-Banderas L., Holgado M.A., Durán-Lobato M., Infante J.J., Álvarez-Fuentes J., Fernández-Arévalo M. (2016). Role of Nanotechnology for Enzyme Replacement Therapy in Lysosomal Diseases. A Focus on Gaucher’s Disease. Curr. Med. Chem..

[23] Qiao H., Hisatomi T., Sonoda K.-H., Kura S., Sassa Y., Kinoshita S., Nakamura T., Sakamoto T., Ishibashi T. (2005). The characterisation of hyalocytes: The origin, phenotype, and turnover. Br. J. Ophthalmol..

