# Supplementary material for: Mass Spectrometry Evaluation of Biomarkers in the Vitreous Fluid in Gaucher Disease Type 3 with Disease Progression Despite Long-Term Treatment
Source: Diagnostics (Basel). 2020 Jan 26;10(2):69. doi: 10.3390/diagnostics10020069 (PMC7168891; doi:10.3390/diagnostics10020069)
Supplement: Supplementary file 1 [file diagnostics-10-00069-s001.pdf]

diagnostics

MDPI

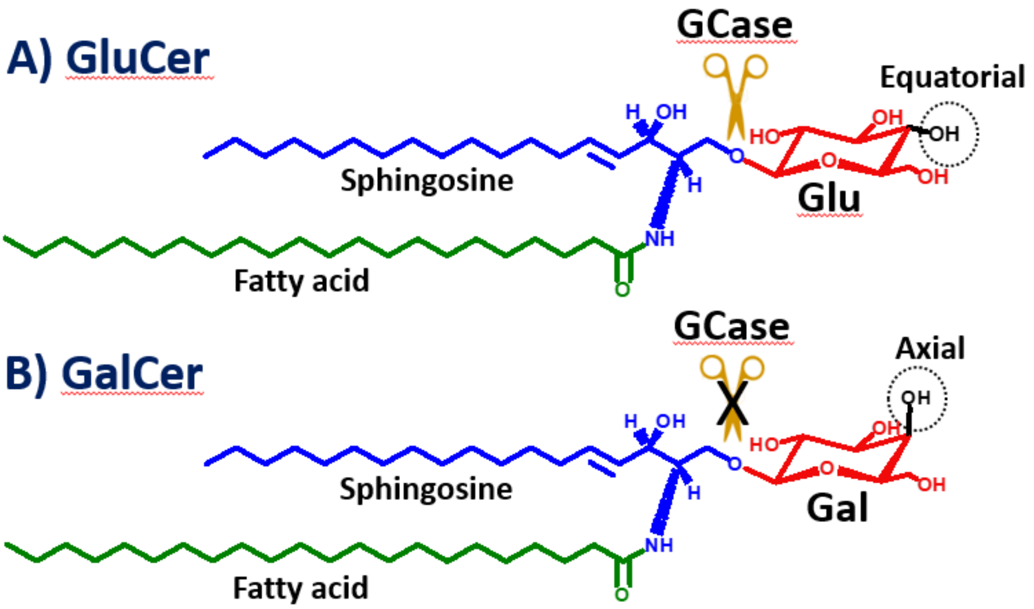

Supplementary Figure S1. Structures of (A) glucosylceramide (GluCer) and (B) galactosylceramide (GalCer) with a C22:0 fatty acid. The glucocerebrosidase (GCase) cleavage site is indicated with scissors on the GluCer molecule. Dotted circles indicate the hydroxyl groups differentiating GluCer and GalCer isomers by their axial or equatorial conformations.
